# Electronic Cognitive Screen Technology for Screening Older Adults With Dementia and Mild Cognitive Impairment in a Community Setting: Development and Validation Study

**DOI:** 10.2196/17332

**Published:** 2020-12-18

**Authors:** Joyce Y C Chan, Adrian Wong, Brian Yiu, Hazel Mok, Patti Lam, Pauline Kwan, Amany Chan, Vincent C T Mok, Kelvin K F Tsoi, Timothy C Y Kwok

**Affiliations:** 1 Department of Medicine and Therapeutics The Chinese University of Hong Kong Hong Kong Hong Kong; 2 Therese Pei Fong Chow Research Centre for Prevention of Dementia The Chinese University of Hong Kong Hong Kong Hong Kong; 3 Jockey Club Centre for Osteoporosis Care and Control The Chinese University of Hong Kong Hong Kong Hong Kong; 4 Gerald Choa Neuroscience Centre, Lui Che Woo Institute of Innovative Medicine The Chinese University of Hong Kong Hong Kong Hong Kong; 5 Jockey Club School of Public Health and Primary Care The Chinese University of Hong Kong Hong Kong Hong Kong

**Keywords:** EC-Screen, cognitive screening, dementia, mild cognitive impairment

## Abstract

**Background:**

A digital cognitive test can be a useful and quick tool for the screening of cognitive impairment. Previous studies have shown that the diagnostic performance of digital cognitive tests is comparable with that of conventional paper-and-pencil tests. However, the use of commercially available digital cognitive tests is not common in Hong Kong, which may be due to the high cost of the tests and the language barrier. Thus, we developed a brief and user-friendly digital cognitive test called the Electronic Cognitive Screen (EC-Screen) for the detection of mild cognitive impairment (MCI) and dementia of older adults.

**Objective:**

The aim of this study was to evaluate the performance of the EC-Screen for the detection of MCI and dementia in older adults.

**Methods:**

The EC-Screen is a brief digital cognitive test that has been adapted from the Rapid Cognitive Screen test. The EC-Screen uses a cloud-based platform and runs on a tablet. Participants with MCI, dementia, and cognitively healthy controls were recruited from research clinics and the community. The outcomes were the performance of the EC-Screen in distinguishing participants with MCI and dementia from controls, and in distinguishing participants with dementia from those with MCI and controls. The cohort was randomly split into derivation and validation cohorts based on the participants’ disease group. In the derivation cohort, the regression-derived score of the EC-Screen was calculated using binomial logistic regression. Two predictive models were produced. The first model was used to distinguish participants with MCI and dementia from controls, and the second model was used to distinguish participants with dementia from those with MCI and controls. Receiver operating characteristic curves were constructed and the areas under the curves (AUCs) were calculated. The performances of the two predictive models were tested using the validation cohorts. The relationship between the EC-Screen and paper-and-pencil Montreal Cognitive Assessment-Hong Kong version (HK-MoCA) was evaluated by the Pearson correlation coefficient.

**Results:**

A total of 126 controls, 54 participants with MCI, and 63 participants with dementia were included in the study. In differentiating participants with MCI and dementia from controls, the AUC of the EC-Screen in the derivation and validation cohorts was 0.87 and 0.84, respectively. The optimal sensitivity and specificity in the derivation cohorts were 0.81 and 0.80, respectively. In differentiating participants with dementia from those with MCI and controls, the AUC of the derivation and validation cohorts was 0.90 and 0.88, respectively. The optimal sensitivity and specificity in the derivation cohort were 0.83 and 0.83, respectively. There was a significant correlation between the EC-Screen and HK-MoCA (*r*=–0.67, *P*<.001).

**Conclusions:**

The EC-Screen is suggested to be a promising tool for the detection of MCI and dementia. This test can be self-administered or assisted by a nonprofessional staff or family member. Therefore, the EC-Screen can be a useful tool for case finding in primary health care and community settings.

## Introduction

Dementia is a global challenge due to the aging population. The prevalence of dementia in older adults ranges from 5% to 7%, and the prevalence of mild cognitive impairment (MCI) ranges from 10% to 20% [1-3]. There are almost 10 million new cases of dementia diagnosed every year worldwide [4]. Studies have shown that early treatment and intervention can help to slow down cognitive decline in older adults [5-7]. The use of a cognitive screening test can facilitate early diagnosis, which in turn helps older adults with dementia and their families to work out a short-term coping and long-term care plan so that they can receive proper dementia-related care, advice, and support in a timely manner, and can live in the community. Improved community support can help to delay or reduce reliance on high-cost residential care services [8].

Paper-and-pencil cognitive screening tests such as the Montreal Cognitive Assessment (MoCA) are commonly used for the detection of cognitive impairment [9]. Although the utility of paper-and-pencil cognitive tests is generally good [10,11], most of these tests must be administered by professional staff, which increases the waiting time for patients and also risks introducing rater biases in test administration and scoring. Moreover, the calculation of the cutoff scores in paper-and-pencil cognitive tests cannot take behavioral data such as response time into account. Furthermore, “practice effects” occur with repeated applications, which could undermine the usefulness of the tests for measuring either the treatment response or the monitoring of disease progression [12,13]. In addition, older adults may not be motivated to seek out or undergo cognitive assessment with health care professionals, or they may have difficulties in accessing health care services. Indeed, a meta-analysis reported a high rate of undetected dementia, especially in China and India [14]. Therefore, it is important to find a way to help family members and health care professionals decide whether it is necessary to seek professional assessment by detecting early signs of cognitive impairment in the older people in their lives or in their care.

Recent studies have proposed the use of digital cognitive tests to overcome some of the above-mentioned barriers, as digital cognitive tests provide automatic, standardized administration procedures, including presentation of the stimulus, scoring, and performance classification [15-18]. Digital cognitive tests can be self-administered or used with minimal assistance by family members or nonprofessional staff, which can significantly increase access to cognitive screening in the general community. Furthermore, digital cognitive tests allow for accurate measurement of participants’ response time, which is known to be affected at an early stage in cognitive disorders [19]. A previous study showed that the diagnostic performance of digital cognitive tests is comparable with that of traditional paper-and-pencil tests [20]. Therefore, digital cognitive tests may play a helpful role as a preliminary screen in the workflow of cognitive assessment; those who show deficits may then undergo further assessment by professionals, thus facilitating better health care resource utilization. However, despite the availability of some commercial digital cognitive tests, their use is not common in Hong Kong, which may be due to the high cost of the tests and language barrier. Therefore, there is a need to develop a brief, user-friendly, and inexpensive digital cognitive test.

To fill this gap, we developed a brief digital cognitive test called the Electronic Cognitive Screen (EC-Screen) using a cloud-based platform that runs on a tablet. The EC-Screen is adapted from the Rapid Cognitive Screen (RCS), which is a short and well-validated paper-and-pencil cognitive test [21]. We aim to promote the use of the EC-Screen in primary health care and community settings in Hong Kong, such as in general practitioner clinics and community elder centers. The objective of this study was to evaluate the validity and performance of the EC-Screen for the detection of MCI and dementia in older adults.

## Methods

### Recruitment of Participants

This study was approved by the clinical research ethics committee of the Chinese University of Hong Kong (CUHK). Participants were recruited from research clinics of the Geriatrics and Neurology divisions of the CUHK, and from a community elderly center, namely the Jockey Club Center for Positive Aging in Hong Kong. The recruitment period was from March to November 2019. The inclusion criteria of the participants were aged ≥55 years, able to communicate in the Chinese language, and adequate perceptual-motor ability so as to be able to participate in cognitive testing. The exclusion criteria were people with uncontrolled psychiatric illnesses and participants who selected the illiterate version. This is because the answer time in the illiterate version is longer than that of the standard version, and therefore we excluded this version for the analysis to enable effective comparison.

Participants with MCI and dementia were consecutive patients from the Geriatrics and Neurology divisions of the CUHK. All of the participants with a diagnosis of MCI and dementia were assessed by a geriatrician. The diagnostic criterion of MCI was based on the Petersen criterion [22], and the diagnostic criterion of dementia was based on the International Classification of Diseases version 10 [23]. The healthy controls were recruited from the Jockey Club Center for Positive Aging and the Division of Neurology of the CUHK. The controls underwent neuropsychological assessment and the Hong Kong version of the MoCA (HK-MoCA) [9,24], and were assessed as cognitively healthy. A purposive sampling method was used [25]. All of the participants provided informed consent through the EC-Screen platform on a designated page that was designed to obtain consent, and the participants also signed a written consent form agreeing to participate in the research.

### EC-Screen

The EC-Screen was developed by the Department of Medicine and Therapeutics and the Division of Neurology of the CUHK. A local software company assisted with software development. The platform reads out all of the questions and then prompts the participant to select the answer on the touchscreen. Modifications of the test instructions were made for any illiterate participant by providing the option for the questions and possible responses to be read out by the software. For participants who are able to read, the platform only reads out the questions and the response choices are shown on the screen. The administration time of the EC-Screen is approximately 5 minutes.

The EC-Screen is composed of two parts. The first part collects the participant’s personal information and the second part is the digital cognitive test. In the personal information part, participants are required to enter their year of birth, gender, education level, and area of residence. In the digital cognitive test part, the participants are required to answer three subtests, including a clock-setting test, story test, and 5-word delayed recognition test. The clock-setting test assesses executive functions and visuospatial abilities, the story test assesses mental flexibility, and the 5-word delayed recognition test assesses memory function. The digital cognitive test part of the EC-Screen was adapted from the RCS [21]. The Chinese Cantonese translation of the RCS was provided by Saint Louis University (St. Louis, MO, USA). The Chinese translation of the test items and the content validity were reviewed by a team of experienced experts, including a geriatrician (TK) and a clinical psychologist (AW). A comparison between RCS and EC-Screen is shown in Multimedia Appendix 1.

In brief, the flow of the digital cognitive test part of the EC-Screen is as follows: At the beginning of the test, the participant is required to learn 5 two-syllable words that are read out by the platform. The participant is then required to answer the clock-setting test in which a clock is presented on the screen and the participant is required to set the clock hands to a specified time. After the clock-setting test, the participant is required to answer the story test, which is a story-based fact conversion test. The platform reads a short story and the participant is required to remember the details of the story and appropriately identify the fact that a well-known landmark belongs to a certain region in Hong Kong. After the story test, the participant is required to answer the 5-word delayed recognition test. A total of 12 two-syllable words containing 5 target words and 7 distractors are presented on the screen, and the participant is asked to indicate which 5 words are the target word that he/she learned at the beginning of the test.

**Figure 1:**
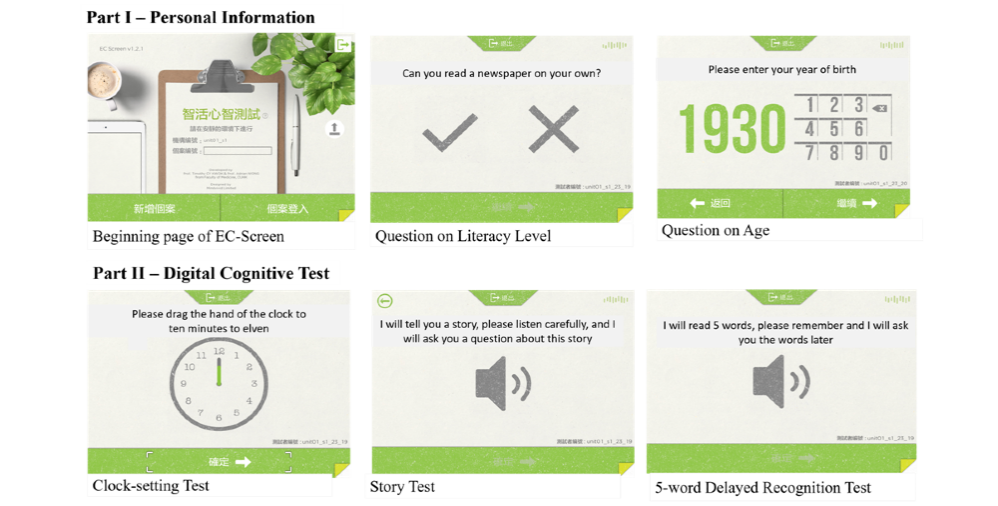
Screenshots of EC-Screen.

The flow of the EC-Screen is shown in Multimedia Appendix 2 and associated screenshots are shown in Figure 1. The total score of the delayed recognition test is 5, and the scoring of the clock-setting test and story test is simply dichotomized as correct or incorrect. The measurement of the time score in each subtest is focused on the interval between the end of the presentation of the instructions and the completion of the task.

### Comparison Test

The HK-MoCA was used as a comparison test. The HK-MoCA is a well-validated multidomain paper-and-pencil cognitive test that assesses visuospatial and executive functions, naming, memory, attention, abstraction, and orientation [9,24]. The maximum total score of the HK-MoCA is 30 and the administration time is approximately 10 to 15 minutes. The EC-Screen and HK-MoCA were administrated by trained research assistants. Both tests were conducted on the same day in the research clinic or elderly center. The assessors were not blind to the participants’ clinical diagnosis during the implementation of the tests because the participants were patients in the clinics. However, since the administration of the EC-Screen is given automatically by the system, the results obtained with the EC-Screen are not affected by this lack of blinding.

### Outcomes

The outcomes were the performance of the EC-Screen in distinguishing the participants with MCI and dementia from controls and in distinguishing the participants with dementia from those with MCI and controls.

### Sample Size Calculation

Based on the estimated prevalence rates of dementia and MCI at 8.9% and 8.5%, respectively [26], with a power of 0.8, a type I error of 0.05, and an expected sensitivity and specificity of 0.80 each, it was determined that a minimal overall sample size of 108 was required in the derivation cohort [27].

### Statistical Analysis

The cohort was randomly split into derivation and validation cohorts (6:4) according to the participants’ disease group (ie, control, MCI, and dementia). In the derivation cohort, multivariable binary logistic regression analysis was performed to test the association between the individual scores and the time spent on each subtest. A list of variables was identified and preliminarily tested using the general linear model. Raw scores and the time spent on each subtest were selected for further testing in the regression model. The scores and time spent on the subtests were standardized to a z-score for analysis. Receiver operating characteristic (ROC) curves were constructed to examine the ability of the predicted probability derived from the optimal logistic regression model with the scores and time spent on the subtests of the EC-Screen used as the explanatory variables. Two predictive models were produced. The first predictive model was used to distinguish participants with MCI and dementia from controls, and the second predictive model was used to distinguish participants with dementia from those with MCI and controls. The areas under the ROC curves (AUCs) were calculated with a 95% CI. A cut-off point was derived at an optimal balance of sensitivity and specificity. The performances of the two predictive models were then tested using the validation cohort. Concurrent validity was evaluated based on the Pearson correlation coefficient between the predicted probability score of the EC-Screen and the total score of the HK-MoCA. A *P* value ≤.05 was regarded as statistically significant. Statistical analyses were conducted using R with the readxl, pROC, and ggpubr packages. 

## Results

### Characteristics of Participants

**Figure 2:**
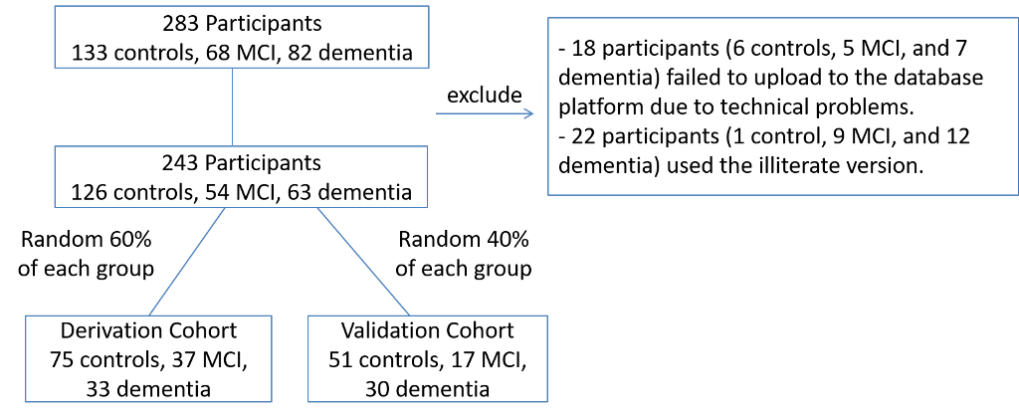
Flow diagram of participants.

A total of 283 participants were recruited for this study. However, data of 18 participants, including 6 controls, 5 with MCI, and 7 with dementia, failed to upload to the database platform due to technical problems. In addition, 22 participants, including 1 control, 9 with MCI, and 12 with dementia, used the illiterate version, and were therefore excluded from the analysis. As a result, the screening results of 243 participants, including 126 controls, 54 participants with MCI, and 63 participants with dementia, were analyzed (Figure 2). The participants were randomly split into the derivation cohort and validation cohort. The characteristics of the participants are shown in Table 1. There were no significant differences in age, gender, educational level, and HK-MoCA score between the derivation and validation cohorts.

**Table 1:** Characteristics of the participants.

Characteristic		Derivation cohort			Validation cohort		
		Control (n=75)	MCI^a^ (n=37)	Dementia (n=33)	Control (n=51)	MCI (n=17)	Dementia (n=30)
Mean age (years), mean (SD)		70.2 (8.1)	76.1 (6.3)	78.6 (7.1)	70.0 (9.3)	78.4 (7.6)	79.8 (7.5)
Females, n (%)		60 (80)	19 (51)	26 (79)	40 (78)	12 (71)	20 (67)
**Education level, n (%)**							
	Primary level or below	29 (39)	18 (49)	18 (55)	13 (25)	12 (71)	23 (77)
	Secondary level or above	46 (61)	19 (51)	15 (45)	38 (75)	5 (29)	7 (23)
HK-MoCA^b^ score, mean (SD)		24.4 (3.3)	20.1 (3.7)	12.2 (6.4)	23.9 (3.9)	19.7 (4.4)	13.8 (5.1)
**Z-score of EC-Screen^c^ subtests, mean (SD)**							
	Clock-setting test	1.24 (1.5)	1.22 (1.5)	0.73 (1.3)	1.41 (1.5)	0.88 (1.4)	1.40 (1.5)
	Delayed recognition test	3.52 (1.4)	2.97 (1.3)	1.45 (1.2)	3.67 (1.4)	2.59 (1.5)	1.43 (1.5)
	Story test	1.08 (1.5)	0.81 (1.4)	0.55 (1.2)	1.18 (1.5)	1.59 (1.5)	0.90 (1.4)
**Time spent on EC-Screen subtests (seconds), mean (SD)**							
	Clock-setting test	31.7 (13.9)	39.6 (16.8)	68.4 (51.3)	31.6 (12.4)	49.4 (27.6)	67.5 (42.0)
	Delayed recognition test	28.0 (12.4)	36.3 (15.7)	43.8 (24.2)	30.3 (14.9)	48.2 (26.2)	55.6 (34.0)
	Story test	75.3 (11.9)	75.8 (18.3)	89.1 (23.4)	73.7 (10.2)	84.7 (13.7)	87.6 (24.3)

^a^MCI: mild cognitive impairment.

^b^HK-MoCA: Hong Kong version of Montreal Cognitive Assessment.

^c^EC-Screen: Electronic Cognitive Screen.

### Validation Results

#### Predictive Model for Distinguishing Participants With MCI and Dementia From Controls

A predicted probability score for having MCI and dementia derived from the EC-Screen was obtained by taking the raw score of the 5-word delayed recognition test and the clock-setting test, as well as the time spent in the 5-word delayed recognition test and clock-setting test into the following regression formula (see Multimedia Appendix 3 for the regression coefficients):

logit (p) = –1.015 – 0.08 (clock score) – 0.68 (delayed recognition score) + 0.03 (clock time) + 0.05 (delayed recognition time)

**Figure 3:**
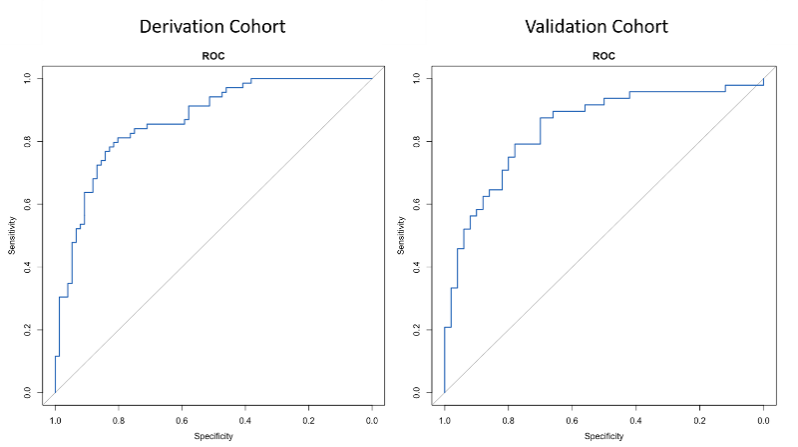
Receiver operating characteristic (ROC) curves of EC-Screen for discriminating among participants with mild cognitive impairment and dementia.

In differentiating participants with MCI and dementia from controls, the AUCs of the EC-Screen in the derivation and validation cohort were high (Table 2). The optimal sensitivity and specificity in derivation cohort were 0.81 and 0.80, respectively, with the cut-off point of the predicted probability score identified as ≥0.43. The sensitivity and specificity in the validation cohort were equivalent but slightly lower. The ROC curves in the derivation cohort and the validation cohort did not show overfitting of the model (Figure 3).

**Table 2:** Results of diagnostic performance of the EC-Screen–derived regression.

Regression model		AUC^a^ (95% CI)	Cut-off value	Sensitivity	Specificity
**Detect MCI^b^ + dementia from controls**					
	Derivation cohort	0.87 (0.81-0.93)	0.43	0.81	0.80
	Validation cohort	0.84 (0.76-0.92)	0.43	0.79	0.78
**Detect dementia from MCI + controls**					
	Derivation cohort	0.90 (0.84-0.95)	0.22	0.83	0.83
	Validation cohort	0.88 (0.81-0.96)	0.22	0.82	0.76

^a^AUC: area under the curve.

^b^MCI: mild cognitive impairment.

#### Predictive Model for Distinguishing Dementia Participants from MCI Participants and Controls

A predicted probability score for having dementia derived from the EC-Screen was obtained by taking the raw score of the 5-word delayed recognition test, the clock-setting test, and the story test, as well as the time spent in the 5-word delayed recognition test and clock-setting test into the following regression formula (see Multimedia Appendix 3 for regression coefficients):

logit (p) = –1.05 – 0.26 (*clock score*) – 0.15 (*story score*) – 0.87 (*delayed recognition score*) + 0.02 (*clock time*) + 0.03 (*delayed recognition time*)

**Figure 4:**
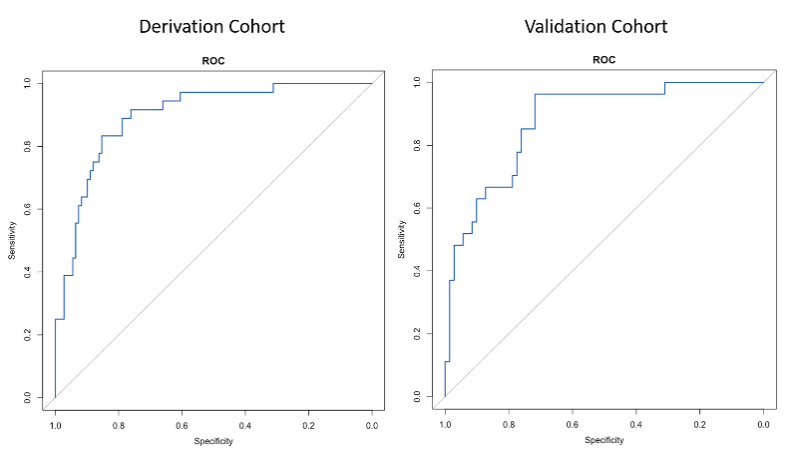
Receiver operating characteristic (ROC) curves of EC-Screen for discriminating among participants with dementia from those with mild cognitive impairment and controls in the derivation and validation cohorts.

In differentiating dementia participants from MCI participants and controls, the AUCs of the EC-Screen of the derivation and validation cohorts were high (Table 2). The optimal sensitivity and specificity in the derivation cohort were 0.83 and 0.83, respectively, with the cut-off point of the predicted probability score identified as ≥0.22. The sensitivity and specificity of the validation cohort were equivalent, but slightly lower (Table 2). The ROC curves in the derivation cohort and the validation cohort did not show overfitting of the model (Figure 4).

### Concurrent Validity

**Figure 5:**
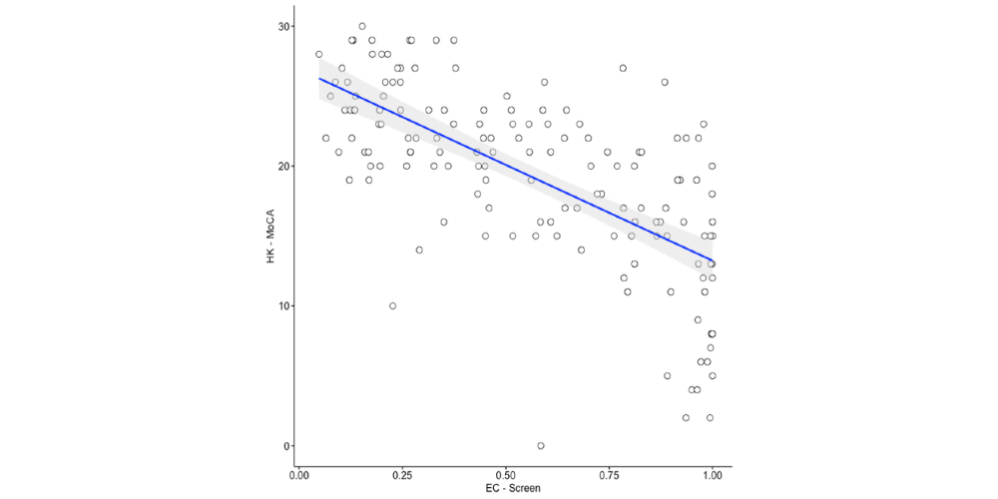
Scatterplot showing the relationship between the predicted probability of EC-Screen–derived regression and the Hong Kong version of the Montreal Cognitive Assessment (HK-MoCA).

There was a significant correlation between the predicted probability score of the EC-Screen–derived regression and the total score of the HK-MoCA (*r*=–0.67, *P*<.001) (Figure 5).

### Administration Process

The average administration time of the EC-Screen was 4.5 minutes. In the qualitative report from users, some of the participants could complete the test by themselves and some of them required assistance from a helper to use the tablet. Therefore, the EC-Screen can be self-administered or assisted by a nonprofessional staff or family member.

## Discussion

### Principal Findings

This study shows that the EC-Screen has good criteria and concurrent validity to identify older adults with MCI and dementia. It is brief and only requires 5 minutes to administer. The automatic administration and scoring algorithm can ease the workload of professional and health care staff. The EC-Screen is a promising tool to use in community centers and primary health care clinics, and thus older adults at risk can receive cognitive screening promptly in the community.

Digital methods allow for capturing response times accurately, which are not easy to capture with paper-and-pencil tests. Participants with MCI and dementia require a longer time in the clock-setting test, which might reflect problems in processing speed and executive functions. The traditional method of the clock-drawing test requires more precise fine motor control, especially when drawing on a tablet. In contrast, the clock-setting test requires less fine motor control and is thus easier for older adults, which minimizes the confounding effects of physical constraints and poor motor dexterity. The diagnostic performance of the clock-setting test was slightly better than that determined for the clock-drawing test in a pilot study [28]. Therefore, the digital clock-setting test was used in the EC-Screen. We used a digital delayed recognition test instead of a traditional method of the delayed free recall test because current voice recognition technology is not yet able to automatically understand a stranger’s voice correctly. Therefore, the digital version of the delayed recognition test was considered to be more practical for use in community settings than the digital version of the delayed free recall test. Delayed recognition tests are commonly used for cognitive screening. Previous studies have shown that the diagnostic performance of the digital version of delayed recognition tests effectively detected both MCI and dementia [20,29]. Both the raw score and time spent in the 5-word delayed recognition test were statistically significant in the regression model, indicating that problems in memory retrieval and processing efficiency are important markers of cognitive disorders. Impairment of delayed memory recognition could reflect problems in encoding, consolidation, or storage, which together comprise a cardinal feature of Alzheimer disease. Therefore, performance on delayed recognition may serve as a more sensitive marker than delayed free recall for identifying patients with early cognitive decline at risk for progression to Alzheimer disease [30]. The question of the story test is not the exact content of the story and requires the participant to convert a fact that a landmark belongs to a given district. Therefore, the story test can assess the mental ability to switch between two concepts.

Some older adults may be aware of their memory decline, and some family members may worry about their parents or grandparents with potential signs of cognitive decline; thus, the EC-Screen can help them to decide whether it is necessary to seek medical and professional advice. Older adults can obtain the cognitive screening assessment from community centers or elderly centers. Therefore, the EC-Screen can promote timely assessment for older adults at risk in the community. 

A digital cognitive test can capture behavioral data accurately. Some studies found that drawing time or drawing process can be a predictive factor for cognitive impairment [31,32]. In the last decade, some multidomain digital cognitive tests have been investigated, including Brain on Track [13] and Computerized Cognitive Screen [33]. These digital cognitive tests assess memory, attention, processing speed, and executive functions, and the administration time is around 20 to 25 minutes. The AUC of Brain on Track for the detection of MCI was 0.86 and the AUC of the Computerized Cognitive Screen for the detection of MCI and dementia was 0.78. There are some commercially available computerized cognitive test batteries such as the Computer assessment for Mild Cognitive Impairment (CAMCI) [34] and CNS Vital Signs [35]. However, the administration time of these tests is longer (>20 minutes) and the costs are rather high. The EC-Screen showed comparable diagnostic performance with a simpler design. Further evaluation of the EC-Screen in a larger cohort of older people recruited from various sources in the community is ongoing. 

Previous studies have reported that the performance on cognitive tests such as the MoCA is affected by education [36-38]. Such education effects are more obvious in Asian countries, as elderly people in these countries are generally less educated [36,38]. The EC-Screen has an adapted version with illiterate options for administration of the test. Twenty-two participants chose the illiterate version in this study. The design of the illiterate version is tailor-made for participants with a very low education level. However, the administration time of this version is longer than that of the standard version, and therefore we excluded the participants who used the illiterate version from this analysis. We are currently planning to separately analyze the participants who took the illiterate version when a larger sample is obtained.

### Limitations

There were some limitations to this study. First, the sample size was small, and therefore EC-Screen results showing that it can discriminate among dementia, MCI, and controls need to be confirmed in a larger study. Second, the test/retest reliability was not investigated in this study and needs to be examined in the future.

### Conclusions

The EC-Screen is suggested to be a promising tool for the detection of MCI and dementia. The EC-Screen is brief and can be self-administered or assisted by a nonprofessional staff or family member. Therefore, it can be a useful tool for case finding in primary health care and community settings.

## Appendix

Multimedia Appendix 1 Comparison of Rapid Cognitive Screen and EC-Screen.

Multimedia Appendix 2 Description of the flow of EC-Screen.

Multimedia Appendix 3 Regression coefficients of the regression models.

## Abbreviations

AUC: area under the curve
CUHK: Chinese University of Hong Kong
EC-Screen: Electronic Cognitive Screen
HK-MoCA: Hong Kong version of the Montreal Cognitive Assessment
MCI: mild cognitive impairment
MoCA: Montreal Cognitive Assessment
RCS: Rapid Cognitive Screen
ROC: receiver operating characteristic
