# Correction: Electronic Cognitive Screen Technology for Screening Older Adults With Dementia and Mild Cognitive Impairment in a Community Setting: Development and Validation Study

**DOI:** 10.2196/26724

**Published:** 2021-01-19

**Authors:** Joyce Chan, Adrian Wong, Brian Yiu, Hazel Mok, Patti Lam, Pauline Kwan, Amany Chan, Vincent C T Mok, Kelvin K F Tsoi, Timothy C Y Kwok

**Affiliations:** 1 Department of Medicine and Therapeutics The Chinese University of Hong Kong Hong Kong China (Hong Kong); 2 Therese Pei Fong Chow Research Centre for Prevention of Dementia The Chinese University of Hong Kong Hong Kong China (Hong Kong); 3 Jockey Club Centre for Osteoporosis Care and Control The Chinese University of Hong Kong Hong Kong China (Hong Kong); 4 Gerald Choa Neuroscience Centre, Lui Che Woo Institute of Innovative Medicine The Chinese University of Hong Kong Hong Kong China (Hong Kong); 5 Jockey Club School of Public Health and Primary Care The Chinese University of Hong Kong Hong Kong China (Hong Kong)

In “Electronic Cognitive Screen Technology for Screening Older Adults With Dementia and Mild Cognitive Impairment in a Community Setting: Development and Validation Study” (J Med Internet Res 2020;22(12):e17332) the authors noted the need to revise the “Acknowledgments” section.

In the originally published article, the Acknowledgments section read as follows:

The development of EC-Screen is supported by The Hong Kong Jockey Club Charities Trust. We greatly appreciate the contributions of the Jockey Club Centre for Positive Ageing in the design and development of the EC-Screen, and Mindvivid Limited for program development of EC-Screen. We also thank Ms. Anthea Ng for her help in data collection and entry.

This section has been revised to:

We thank Professor JE Morley, Saint Louis University School of Medicine, USA, for having agreed to let us adapt his Rapid Cognitive Screen test into EC-Screen for older Chinese people. We are also grateful for the funding support from the Hong Kong Jockey Club Charities trust, and for the support of the Jockey Club Centre for Positive Ageing for its contribution in design and data collection. We appreciate the support from Mindvivid Limited in software development of EC-Screen. We also thank Ms. Anthea Ng, research assistant of the Division of Neurology at The Chinese University of Hong Kong, for her help in data collection and entry.

The correction will appear in the online version of the paper on the JMIR Publications website on January 19, 2021, together with the publication of this correction notice. Because this was made after submission to PubMed, PubMed Central, and other full-text repositories, the corrected article has also been resubmitted to those repositories.

