# Supplementary material for: Electronic Cognitive Screen Technology for Screening Older Adults With Dementia and Mild Cognitive Impairment in a Community Setting: Development and Validation Study
Source: J Med Internet Res. 2020 Dec 18;22(12):e17332. doi: 10.2196/17332 (PMC7775823; doi:10.2196/17332)
Supplement: Multimedia Appendix 1 [file jmir_v22i12e17332_app1.docx]

Comparison of Rapid Cognitive Screen and EC-Screen

|  | **Rapid Cognitive Screen** | **EC-Screen** |
| --- | --- | --- |
| **Format** | Paper-and-Pencil | Digital and run on a tablet |
| **Administration Time** | 5 minutes | 5 minutes |
| **Test Items** | Clock-drawing Test | Clock-setting Test |
|  | Story Test | Story Test |
|  | 5-Word Delayed Free Recall Test | 5-Word Delayed Recognition Test |
