# Supplementary material for: Electronic Cognitive Screen Technology for Screening Older Adults With Dementia and Mild Cognitive Impairment in a Community Setting: Development and Validation Study
Source: J Med Internet Res. 2020 Dec 18;22(12):e17332. doi: 10.2196/17332 (PMC7775823; doi:10.2196/17332)
Supplement: Multimedia Appendix 2 [file jmir_v22i12e17332_app2.docx]

| **Flow of the EC-Screen** | |  |  |
| --- | --- | --- | --- |
| 1. | Obtain consent |  |  |
| **Part I Personal Information** | |  |  |
| 2. | Select literacy level |  |  |
| 3. | Input age |  |  |
| 4. | Select gender |  |  |
| 5. | Select education level |  |  |
| 6. | Select district of residence |  |  |
| **Part II Digital Cognitive Test** | | **Scoring** | **Time Score** |
| 7. | Learning of 5 target words | -- | -- |
| 8. | Clock-setting test | Correct/  Incorrect | From the end of the presentation of the instruction to the completion of clock setting |
| 9. | Story test | Correct/  Incorrect | From the end of the question to finish selection of answer |
| 10. | 5-word delayed recognition test | Max score: 5 | From the end of the presentation of the instruction to finish selection of the words |
