# Supplementary material for: Electronic Cognitive Screen Technology for Screening Older Adults With Dementia and Mild Cognitive Impairment in a Community Setting: Development and Validation Study
Source: J Med Internet Res. 2020 Dec 18;22(12):e17332. doi: 10.2196/17332 (PMC7775823; doi:10.2196/17332)
Supplement: Multimedia Appendix 3 [file jmir_v22i12e17332_app3.docx]

Regression coefficients of the regression models

|  | **Beta** | **S.E.** | ***P*-value** |
| --- | --- | --- | --- |
| 1. **Detect MCI + Dementia from Control** | | | |
| Clock-setting Test Score | -0.08 | 0.14 | .57 |
| Delayed Recognition Test Score | -0.68 | 0.16 | <.001 |
| Clock-setting Test Time | 0.03 | 0.01 | .04 |
| Delayed Recognition Test Time | 0.05 | 0.02 | .001 |
| 1. **Detect Dementia from MCI + Control** | | | |
| Clock-setting Test Score | -0.26 | 0.18 | .15 |
| Story Test Score | -0.15 | 0.19 | .42 |
| Delayed Recognition Test Score | -0.87 | 0.18 | <.001 |
| Clock-setting Test Time | 0.02 | 0.01 | .03 |
| Delayed Recognition Test Time | 0.03 | 0.01 | .02 |

Abbreviations: Beta, parameter estimate; S.E., standard error.
